# The distribution of Ohio’s Certificates to Recommend: who will “prescribe” medical marijuana?

**DOI:** 10.1186/s42238-020-00019-z

**Published:** 2020-02-28

**Authors:** Frederic Stuart Leeds, Ryan K. Levinthal, Morgan T. Alexander, Timothy N. Crawford

**Affiliations:** 1 Department of Family Medicine, Wright State University Boonshoft School of Medicine, Dayton, OH USA; 2 Department of Population and Public Health Sciences, Wright State University Boonshoft School of Medicine, Dayton, OH USA

**Keywords:** Ohio, Medical marijuana, Certificate to recommend, Age, Medical degree, Specialty, Gender, Access to care

## Abstract

**Background:**

Under Ohio Medical Marijuana Control Program rules, Ohio physicians that recommend medical marijuana (MMJ) to patients must possess a Certificate to Recommend (CTR) from the State Medical Board. Although a pre-program state survey indicated that more than a quarter of Ohio physicians were likely to recommend MMJ, only 473 physicians obtained CTRs in the first year of the program, amounting to just 1.39% of the physician workforce. The purpose of this study is to evaluate demographic factors that influence a physician’s decision to obtain the CTR.

**Method:**

Using physician demographic data extracted from Ohio’s databases of medical licensees and CTR holders, as well as the American Medical Association Physician Masterfile, prevalence ratios for CTR holders were calculated for specialty, medical degree (Doctor of Medicine, MD, vs. Doctor of Osteopathy, DO), age and gender. A multivariate model was implemented to generate adjusted prevalence ratios (aPRs) reflecting the independent effects of specialty, degree, and age. To assess temporal variations in CTR acquisition, per-specialty CTR counts were also plotted as a function of program month.

**Results:**

The best-represented specialties among CTR holders were Family Medicine (29.11%), Internal Medicine and its subspecialties (22.57%), and Anesthesiology (9.07%). Expressed as an adjusted per-specialty prevalence ratio in reference to Family Medicine, the dominant specialty was Physical Medicine and Rehabilitation (aPR 2.08, 95% CI 1.34–3.24), with the lowest measurable prevalence ratios found in Pediatrics (aPR 0.17, 95% CI 0.10–0.30) and Surgery (aPR 0.33, 95% CI 0.22–0.50). DOs were more likely to obtain CTRs than MDs (aPR 1.72, 95% CI 1.39–2.15). The mean age of CTR holders was 54.03 +/− 11.43, vs. 51.13 +/− 13.38 for non-CTR holders (*p* < .0001). Although gender could not be included in the multivariate model, males were more likely than females to obtain a CTR (PR **1.54, 95%CI 1.26–1.89).** A plot of per-month CTR acquisition by specialty demonstrated a fairly consistent specialty distribution of CTRs in the first year, as well as variations in overall CTR acquisition that may correspond to program-operational events.

**Conclusion:**

Specialty*,* type of medical degree, and age all correlate independently with the likelihood of registering to recommend medical marijuana in Ohio. Specialty distribution of CTRs remained fairly consistent in the program’s first year, although overall CTR acquisition may be sensitive to program-operational events such as delays in dispensary opening or product availability.

## Introduction

Thirty-three US states and the District of Columbia have legalized marijuana and marijuana products for medical use (Laws 2019). Another thirteen states permit the use of cannabidiol (CBD) under limited circumstances. The first medical marijuana (MMJ) program was instituted in California in 1996, followed 2 years later by programs in Alaska, Oregon, and Washington (Bridgeman and Abazia 2017). By 2016, a majority of states had legalized MMJ, and in June of that year, HB 523 passed in Ohio (House Bill 523 2016), thereby establishing the Ohio Medical Marijuana Control Program (OMMCP). The OMMCP is typical of state medical cannabis programs in that it includes such features as a patient registry and a defined list of qualifying diagnoses for which a physician may “prescribe” MMJ (as cannabis is still an illegal Schedule I substance, the term “recommend” is preferred (Bridgeman and Abazia 2017). Ohio is one of a minority of states that registers its participating physicians (State-by-State Legal Information and Forms for Recommending Cannabis 2019), and it is unique in requiring physicians to obtain a license endorsement termed a *Certificate to Recommend (CTR) (*OAC 4731-32-02 Certificate to recommend medical marijuana 2016*)*.

From the outset, the OMMCP has been beset by numerous obstacles and difficulties. Although HB 523 mandated that an electronic physician CTR registry was to be implemented by the State Medical Board on or before September 8, 2017, the registry was not operational until April 2018. Dispensaries did not begin opening until January 2019, and by the end of the first year of CTR registration, there were only 18 operational dispensaries serving Ohio’s 88 counties (Ohio Medical Marijuana Control Program Advisory Committee Meeting (April Report) 2019). Similar delays have been encountered with respect to the due diligence and certification process for Ohio cannabis cultivators and processors (Associated Press 2019).

Physician engagement with the OMMCP has been equally sluggish. A 2017 pre-program survey conducted by the Ohio State Medical Board (Ohio Resurvey after Draft Rule 2017) found that more than 25% of Ohio physicians considered themselves “likely” or “very likely” to recommend MMJ to their patients. This initial level of support has not, however, translated into a comparable degree of physician participation in the program. CTR registration went live in April 2018, but physician enrollment to date remains under 2% (Program Update 2019).

Although low rates of physician participation have been observed in other state medical cannabis programs (Sideris et al. 2018; Aggarwal et al. 2009), little is known about the factors that influence a physician’s decision to become certified or registered to recommend MMJ. Regarding physician knowledge and attitudes, surveys have identified consistent themes such as: inadequate training in the therapeutic use of cannabis, insufficient evidence regarding benefits and harms, and concerns regarding misuse and abuse (Sideris et al. 2018; Kondrad and Reid 2013; Michalec et al. 2015; Philpot et al. 2019; Carlini et al. 2017). However, these studies have generally relied on voluntary survey methodologies, with a focus on physicians’ perceptions and self-reported biases.

In this study, we propose to assess objective physician factors that are associated with obtaining a CTR in Ohio. Demographic variables such as gender and practice specialty have been shown to correlate with physician views of medical cannabis (Ebert et al. 2015; Gardiner et al. 2019; Charuvastra et al. 2005), and in a broader sense, with many kinds of attitudes and behaviors among clinicians (Tsugawa et al. 2017; Santos et al. 2016; Antiel et al. 2013). We therefore hypothesize that a decision to obtain the CTR, and therefore the likelihood that a physician intends to recommend marijuana therapeutically, will be influenced by such variables as specialty, age, and gender, as well as type of medical degree (MD vs. DO). As the Ohio program is still early in its development, and is therefore still negotiating organizational and logistical obstacles, we further hypothesize that CTR uptake may vary over time, with a chronology reflecting both “early adopters” and steady growth after program establishment.

As the CTR requirement may prove to be a barrier to access to care with respect to MMJ, a deeper understanding of the determinants of CTR uptake is clearly needed.

## Methods

The OMMCP CTR Roster, effective May 9, 2019, was obtained from the program website (Ohio State Medical Board 2019). This was cross-referenced against the full State Medical Board licensee roster (accessed at the same website), in order to identify specialty, gender, and degree type. To calculate prevalence ratios for these variables, physician demographic data was obtained from the most recent version of the Association of American Medical Colleges Ohio Physician Workforce Profile (2017 State Physician Workforce Data Book 2017), which in turn was derived from the American Medical Association Physician Masterfile. This file provided counts on specialty and gender. In addition, the merged OMMCP CTR Roster/State Medical Board licensee roster was used to examine prevalence of CTR as this file had raw data on age, specialty, and degree type. The two files (i.e., merged dataset and workforce profile) were compared to examine any differences in physician counts and prevalence of CTR.

To simplify analysis, Internal Medicine subspecialties such as gastroenterology and cardiology, the CTR counts for which were generally small, were grouped with general Internal Medicine. Similarly, surgical subspecialties were grouped under the general heading of Surgery. In addition, all remaining specialties with very low CTR counts (< 10) were grouped together to create an ‘Other’ category.

### Analysis

Descriptive statistics were conducted to describe the CTR holders in Ohio with frequencies and percentages for all categorical variables, and means and standard deviations for all continuous variables. Prevalence (per 100 active physicians) was calculated for all Ohio physicians, and for each group (e.g., sex, degree, and specialty) and was presented as percentages. Prevalence, defined as the number of physicians with a CTR divided by the number of total Ohio physicians, was calculated using data from the Association of American Medical Colleges Ohio Physician Workforce Profile and the State Medical Board licensee roster. To examine associations between acquisitions of CTR and certain characteristics (e.g., age, sex, degree, and specialty), χ^2^ tests were conducted for categorical variables and independent t-tests were conducted for the continuous variable. To examine characteristics associated with obtaining a CTR, modified Poisson regression, with robust error variance, was conducted to obtain crude and adjusted prevalence ratios along with 95% confidence intervals. Prevalence ratios were defined as the ratio of the proportion of CTR holders for one group over the proportion of CTR holders for another group (e.g., proportion of CTR holders for males/proportion of CTR holders for females). The adjusted regression model included age, degree, and specialty. Gender was not included as the merged dataset did not include gender. As Family Medicine comprised the largest absolute number of CTR holders, it was used as the reference standard for the calculation of specialty prevalence ratios. Data were analyzed using SAS version 9.4 (Cary, NC) and Excel and an alpha of .05 was used.

## Results

Between April 2018 and April 2019, 474 physicians (1.39%) in Ohio received their CTRs. Table 1 presents the characteristics for those who received their CTR. The majority of CTR holders were male (74.26%) and MDs (70.25%). Family medicine (29.11%) and internal medicine (22.57%) had the two highest prevalences among the different specialties.

**Table 1:** Characteristics among all CTR holders in Ohio (*N* = 474)

	n (%)
Overall	474 (1.39)
Age – mean (sd)	54.03 (11.43)
Sex	
Female	122 (25.74)
Male	352 (74.26)
Degree	
MD	333 (70.25)
DO	141 (29.75)
Specialty	
Family Medicine	138 (29.11)
Anesthesiology	43 (9.07)
Emergency Medicine	27 (5.70)
Internal Medicine^a^	107 (22.57)
Neurology	21 (4.43)
OB/GYN	18 (3.80)
PM&R	22 (4.64)
Pediatrics	14 (2.95)
Psychiatry	38 (8.02)
Surgery^b^	31 (6.54)
Other^c^	15 (3.16)

*OB/GYN* Obstetrics and Gynecology

*PM&R* Physical Medicine and Rehabilitation

^a^Includes Internal Medicine and subspecialties

^b^Includes general surgery and surgery specialties

^c^Includes all other specialties

Table 2 compares the prevalence (expressed as percentages) between the Ohio Physician Workforce Profile and the Ohio Physician Roster. Males (1.58%) compared to females (1.02%) had a higher prevalence of CTR holders. DOs (2.85%) had a higher prevalence of CTR holders compared to MDs (1.13%). For specialty, the prevalence was similar for both data sets. The prevalence of CTR holders was the highest for the Physical Medicine and Rehabilitation (PM&R) specialty.

**Table 2:** Prevalence (per 100 active physicians) of CTR by sex, degree, and specialty comparing the Ohio Physician Workforce Profile and the Ohio Physician Roster file

	Ohio Physician Workforce Profile (*N* = 34,217)	State Medical Board Licensee Roster (*N* = 34,400)
	% (ctr/n)	% (ctr/n)
Overall	1.39 (474/34217)	1.38 (474/34400)
Sex*		
Female	1.02 (122/11928)	–
Male	1.58 (352/22289)	–
Degree ^†^		
MD	–	1.13 (333/29458)
DO	–	2.85 (141/4942)
Specialty		
Family Medicine	3.26 (138/4231)	3.44 (138/4007)
Anesthesiology	3.04 (43/1414)	2.66 (43/1614)
Emergency Medicine	1.61 (27/1681)	1.76 (27/1530)
Internal Medicine^a^	1.36 (107/7886)	1.35 (107/7948)
Neurology	4.31 (21/487)	3.03 (21/693)
OB/GYN	1.23 (18/1459)	1.32 (18/1367)
PM&R	6.47 (22/340)	6.06 (22/363)
Pediatrics	0.67 (14/2085)	0.47 (14/2970)
Psychiatry	3.33 (38/1142)	3.20 (38/1188)
Surgery^b^	1.24 (31/2491)	1.14 (31/2721)
Other^c^	0.14 (15/11001)	0.15 (15/9999)

*Sex was not available in the Ohio Physician Roster file

^†^Degree was not available in the Ohio Physician Workforce Profile

*OB/GYN* Obstetrics and Gynecology

*PM&R* Physical Medicine and Rehabilitation

^a^Includes Internal Medicine and subspecialties

^b^Includes general surgery and surgery specialties

^c^Includes all other specialties

Table 3 examines the bivariate associations between receipt of CTR and characteristics of the physicians. There were significant associations between receipt of CTR and age, degree, and specialty. Physicians with a CTR were older compared to those without a CTR (54.03 versus 51.13, *p* < .0001). CTR holders were more likely to be in the family medicine, anesthesiology, PM&R, and psychiatry specialties compared to non-CTR holders.

**Table 3:** Comparing CTR acquisition by Age, Degree, and Specialty using the Ohio Physician Roster (*N* = 34,400)

	CTR		p*
	Yes	No	
Age – mean (sd)	54.03 (11.43)	51.13 (13.38)	<.0001
Degree	n(%)	n(%)	<.0001
MD	333 (70.25)	29,125 (85.85)	
DO	141 (29.75)	4801 (14.15)	
Specialty			<.0001
Family Medicine	138 (29.11)	3869 (11.40)	
Anesthesiology	43 (9.07)	1571 (4.63)	
Emergency Medicine	27 (5.70)	1503 (4.43)	
Internal Medicine^a^	107 (22.57)	7841 (23.11)	
Neurology	21 (4.43)	672 (1.98)	
OB/GYN	18 (3.80)	1349 (3.98)	
PM&R	22 (4.64)	341 (1.01)	
Pediatrics	14 (2.95)	2956 (8.71)	
Psychiatry	38 (8.02)	1150 (3.39)	
Surgery^b^	31 (6.54)	2693 (7.94)	
Other^c^	15 (3.16)	9981 (29.42)	

*Tests conducted were t-test for continuous variable and χ^2^ for categorical variables

*OB/GYN* Obstetrics and Gynecology

*PM&R* Physical Medicine and Rehabilitation

^a^Includes Internal Medicine and subspecialties

^b^Includes general surgery and surgery specialties

^c^Includes all other specialties

Table 4 presents the crude (unadjusted) and adjusted prevalence ratios. Controlling for age, and specialty, degree was significantly associated with receipt of CTR. Receipt of CTR was 1.72 (95% Confidence Interval (CI) = 1.39–2.15) times higher for DOs compared to MDs. Controlling for age and degree, specialty was significantly associated with the receipt of CTR. Compared to the family medicine specialty, physicians in the PM&R specialty were more likely to receive their CTR (prevalence ratio = 2.08; 95% CI = 1.34–3.24).

**Table 4:** Crude and Adjusted Prevalence Ratios using the Ohio Physician Roster (*N* = 34,400)

	PR (95% CI)	aPR (95% CI)
Age (per one-year increase)	**1.02 (1.01–1.02)**	**1.01 (1.01–1.02)**
Sex*		
Male	**1.54 (1.26–1.89)**	–
Female	Ref	–
Degree		
MD	Ref	Ref
DO	**2.52 (2.08–3.07)**	**1.72 (1.39–2.15)**
Specialty		
Family Medicine	Ref	Ref
Anesthesiology	0.77 (0.55–1.08)	0.87 (0.61–1.23)
Emergency Medicine	**0.51 (0.34–0.77)**	**0.52 (0.33–0.80)**
Internal Medicine^a^	**0.39 (0.30–0.50)**	**0.44 (0.34–0.58)**
Neurology	0.88 (0.56–1.38)	0.96 (0.60–1.55)
OB/GYN	**0.38 (0.23–0.62)**	**0.42 (0.25–0.69)**
PM&R	**1.76 (1.14–2.72)**	**2.08 (1.34–3.24)**
Pediatrics	**0.14 (0.08–0.24)**	**0.17 (0.10–0.30)**
Psychiatry	0.93 (0.65–1.32)	1.06 (0.73–1.53)
Surgery^b^	**0.30 (0.20–0.45)**	**0.33 (0.22–0.50)**
Other^c^	**0.05 (0.03–0.09)**	**0.06 (0.03–0.10)**

*PR* Prevalence Ratio

*aPR* Adjusted Prevalence Ratio

*CI* Confidence Interval

*Sex was not included in the regression analysis as it was not a variable in the Ohio Physician Roster data set

*OB/GYN* Obstetrics and Gynecology

*PM&R* Physical Medicine and Rehabilitation

^a^Includes Internal Medicine and subspecialties

^b^Includes general surgery and surgery specialties

^c^Includes all other specialties

Note: Bolded numbers represent prevalence ratios significant at the .05 level

**Fig. 1:**
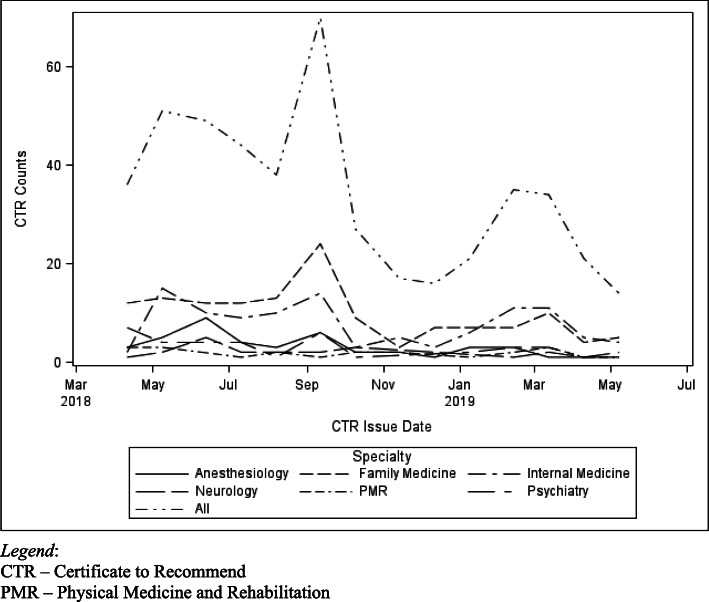
Certificates to Recommend: Counts Per Month, Total and by Specialty. CTR – Certificate to Recommend. PMR – Physical Medicine and Rehabilitation

Fig. 1 shows the number of new CTRs by specialty and month of issue for the six specialties with the highest overall per-specialty CTR prevalences. CTR acquisition was dominated by Family Medicine and Internal Medicine across the study interval, with Family Medicine physicians obtaining the largest raw numbers of CTRs in most months. For most specialties, peaks in CTR acquisition occurred in the first 3 months of program operation, but also in bands around September 2018 and March 2019. A steep drop-off was also observed after the September 2018 peak, spanning the months of October–December 2018.

## Discussion

### Prevalence studies

Medical marijuana has been nominally legal in Ohio since September 8, 2016. However, physician engagement, as reflected in rates of CTR acquisition, has been surprisingly weak. This contrasts with pre-program surveys that predicted much more robust support (Ohio Resurvey after Draft Rule 2017; Medical Marijuana Physician Survey 2016). The explicit purpose of the present study was not to explain this discrepancy, but simply to assess the demographic factors that influence physician participation in the OMMCP. The data we present here support the assertion that specialty, medical degree, and age are independently correlated with the decision to obtain credentials to recommend MMJ.

Specialty was expected to be a strong predictor of CTR acquisition. A national questionnaire study from 2005 suggested that OB/Gyn and Internal Medicine physicians were more supportive of marijuana as medical therapy than either Family Medicine physicians or psychiatrists (Charuvastra et al. 2005), although these results were not statistically significant. In the aforementioned New York study (Sideris et al. 2018), it was reported that one-third of registered physicians elected to identify themselves on a public website, and of these, the overwhelming majority consisted of Primary Care and Pain Medicine practitioners. In a small survey of licensed Delaware physicians (who may recommend MMJ without any form of registration or certification), a very different sort of distribution was described, with Emergency Medicine physicians and pediatricians reporting a higher likelihood of recommending MMJ than Primary Care physicians (Michalec et al. 2015). The present study differs from the foregoing reports in that, rather than relying on physician surveys or voluntary reporting, it combines the use of a comprehensive, non-voluntary state database with an objective outcome variable, i.e., CTR acquisition. Despite these methodological differences, some meaningful comparisons to prior reports are possible, if CTR acquisition is taken, at a minimum, to be an indicator of support for MMJ. Indeed, the Ohio State Board of Pharmacy has recently announced that, as of September 2019, 60.2% of CTR holders have issued at least one MMJ recommendation (Miller 2019); clearly the CTR signals both support, and to some extent, an intention to treat with MMJ.

We observe that CTR acquisition is indeed highly specialty-dependent, with a distribution distinct from previous reports in the literature. Based on absolute numbers, Family Medicine and Internal Medicine physicians comprise the majority of CTR holders. However, these specialties collectively represent a plurality of Ohio physicians, and per-specialty CTR prevalence ratios were comparable for Family Medicine, Neurology, Anesthesiology, and Psychiatry. The highest prevalence was found for Physical Medicine and Rehabilitation (PM&R), while the lowest prevalences were noted for Surgery, OB/Gyn, and Pediatrics, and Other. For a small group of specialties (e.g., Dermatology, Radiology, Pathology), very few (0–5) CTRs were acquired.

With respect to medical degree, DO physicians were two to three times more likely than MD physicians to obtain CTRs. This may partly be explained by specialty factors: among CTR holders with the DO degree, a majority were in Family Medicine or Internal Medicine – the specialties with the highest gross numbers of CTRs. However, our multivariate analysis clearly demonstrates a “DO effect” that holds across specialties. To our knowledge, this is the first report in the literature of a difference in MMJ program enrollment between physicians with MD and DO degrees.

Physician age was expected to be another significant demographic factor influencing physician engagement in MMJ programs. In Colorado, a survey of family physicians reported that those between the ages of 20–29 were less likely than other age groups to issue a MMJ recommendation (Kondrad and Reid 2013). In the small cross-sectional study of Delaware physicians, there was no relationship between age and likelihood of recommending; there were, however, differences observed between groups with different levels of practice experience – such that physicians with 11–20 years of experience were most likely to issue recommendations (Michalec et al. 2015). Our study found that physician age correlated independently with CTR acquisition, with CTR holders tending to be, on average, almost 3 years older than non-CTR-holders.

Gender may also be a factor in predicting support for MMJ as well as likelihood of obtaining credentials to recommend it. Although an older US study showed no difference in MMJ attitudes between male and female physicians (Charuvastra et al. 2005), a more recent Israeli survey did find greater support among male physicians (Ebert et al. 2015). A 2018 survey of 167 New York state physicians found no gender differences between registered and non-registered physicians (Sideris et al. 2018). A gender effect was also observed in our data, with male physicians more than 50% more likely to have acquired a CTR than their female colleagues. As gender could not be included in the multivariate analysis, it is not possible to state with certainty that the gender effect is independent of specialty, medical degree. or age. However, given the relatively strong representation of women among the specialties with high CTR counts, as well as among DO physicians, gender is very unlikely to have interacted strongly with specialty or degree in this study. On the other hands, an interaction between gender and age is possible, particularly given that female physicians in the US are younger, on average, than their male counterparts (Young et al. 2019).

The reasons for the specialty, degree, age, and gender distributions reported here were not explicitly addressed in this study. We might speculate that specialty exerts its primary influence as a function of the likelihood of encountering patients with qualifying diagnoses, although specialty-correlated personal and political views, as well as the positions of official organizations (AAP COMMITTEE ON SUBSTANCE ABUSE COA 2015; John et al. 2018; American Psychiatric Association 2018; AAFP 2018) may also play a role. Certainly, the lower prevalences observed for OB/Gyn and Pediatrics are at least in partly attributable to apprehension about using MMJ in sensitive populations – although this seems to contradict findings in previous reports (Michalec et al. 2015; Charuvastra et al. 2005).

More work is needed to help understand the rather more complex questions surrounding specialty differences in CTR acquisition. Initial hypotheses should focus on profiling CTR holders vs. non-holders, exploring the relative likelihoods and experiences of treating patients with qualifying diagnoses, and assessing the knowledge, beliefs, attitudes, and reasoning that drive the CTR decision.

The reasons for the observed DO-MD disparity are likewise unclear. There are no published studies elucidating differences between MDs and DOs with regard to MMJ knowledge, behavior or atttudes. DOs do appear to have more favorable positions on “non-mainstream therapies” (Johnson and Kurtz 2002), but it is unclear whether this translates into a higher level of acceptance of MMJ, or greater willingness to recommend it to patients. Further studies are needed to explore the underlying attitudinal and practice differences that form the basis for the observed “DO effect” on physician MMJ registration.

The finding of age as a positive correlator with CTR acquisition was somewhat surprising to us. The effect, though small, runs counter to the perception that younger physicians will be, in reflecting age-dependent atttitudes toward marijuana in general (Pacek et al. 2015), more favorably disposed toward engagement in an MMJ program. The reasons underlying this finding are likely to be complex. Younger physicians may be less confident about embracing controversial therapies, and more fearful about peer disapproval and perceived threats to their reputations. This is likely counterbalanced, albeit incompletely, by increased conservatism and negative generational attitudes about MMJ among older physicians. The age effect may, however, be a surrogate for experience and time in service, as no age differences in the attitudes toward MMJ as legitimate therapy were observed in a study of Colorado medical students (Chan et al. 2017). And as noted, the potential interaction of age and gender must also be considered. Clearly more work is needed to understand the nuanced factors that form the basis of the effect of age on physician MMJ engagement.

We might speculate that the male-to-female gender predominance among CTR holders relates to gender-specific differences in political or personal views, although female physicians are more likely than male physicians to identify as moderate or liberal (Frank 1999). Female physicians, on the other hand, may be more likely to adhere to evidence-based practice guidelines (Tsugawa et al. 2017), which are notably lacking for most MMJ indications (National Academies of Sciences E, Medicine 2017). These observations might form the basis for further gender-based research exploring the motivations and attitudes of CTR holders vs. non-holders.

### Time course study

Because we are evaluating demographic factors associated with CTR uptake in the OMMCP’s first year, a reasonable question arises as to whether the findings are representative and generalizable to a more mature and established MMJ program. We also wanted to answer ancillary questions regarding the effects of specific program events on CTR uptake, such as the prolonged delay in the opening of operational dispensaries. A similar chronological analysis was described by Sideris et al. (Sideris et al. 2018), noting that physician and patient registration accelerated after a key program event, i.e., the addition of “chronic pain” as a qualifying condition. In Fig. 1, we observe a pattern of brisk initial CTR uptake that peaks in May and diminishes through August 2018. A second, short-lived peak occurred in September, possibly coinciding with the planned openings of the first dispensaries. However, delays in dispensary certification, product delivery and patient registration (Gillispie 2019) pushed back the first dispensary opening to January 2019, at which time another CTR acquisition peak was observed. Rates of CTR uptake declined to < 20 per month after that, and according to the most recent OMMCP program update (Program Update 2019), they appear to remain at that level. We also note in Fig. 1 that, with few exceptions, the specialty CTR counts track proportionally with the total CTRs per month, suggesting that the relative specialty distributions observed in the first year represent a stable and consistent pattern that might persist in subsequent years.

### Limitations

Although the Ohio physician roster maintained by the State Medical Board included such fields as medical degree and specialty, it did not identify gender; therefore, our gender analysis was limited to the aggregate denominators by specialty appearing in the Ohio Physician Workforce document. This also precluded the inclusion of gender in the multivariate analysis. We have noted the aspects of the study that are potentially impacted by this limitation elsewhere in this discussion. In addition, the specialty data are voluntarily reported to the board by physicians, so it is difficult to exclude inaccuracies in the calculation of specialty prevalences — though this source of error is likely to be small. It is also worth reiterating that we elected to group medical and surgical subspecialties within the larger categories of their parent specialties. There certainly was heterogeneity noted in the CTR uptake between, for example, medical subspecialties such as Pulmonology and Hematology-Oncology. However, the absolute numbers for these subgroups were small, and would not substantially alter the core observations and conclusions presented here.

## Conclusions

This study is, to our knowledge, the first to report and analyze a comprehensive dataset of state-reported demographics of physician registration in the first year of a medical cannabis program. We find that specialty, type of medical degree, and age all correlate independently with the likelihood of obtaining a CTR for medical marijuana in Ohio. A correlation with gender may also exist, but this necessitates further evaluation. A chronological analysis demonstrated that physician registration may be sensitive to program-operational events such as delays in dispensary opening or product availability; it also provided some support for the cautious prediction that the specialty distribution of CTRs would remain relatively consistent after the first year. Future studies are needed to improve our understanding of the underlying reasons for the specialty, degree, age and gender effects on physician registration in MMJ programs.

## Data Availability

The datasets supporting the conclusions of this article are available in the online resources maintained by the Ohio State Medical Board (Ohio State Medical Board 2019) and the Association of American Medical Colleges (2017State Physician Workforce Data Book 2017).

## Abbreviations

aPR: Adjusted Prevalence Ratio (for the multivariate model)
CI: Confidence Interval
CTR: Certificate to Recommend
DO: Doctor of Osteopathic Medicine
HB 523: Ohio House Bill 523
MD: Doctor of Medicine (Allopathic)
MMJ: Medical Marijuana
OB/Gyn: Obstetrics/Gynecology
OMMCP: Ohio Medical Marijuana Control Program
PM&R: Physical Medicine and Rehabilitation
PR: Prevalence Ratio
